# Quantitative Evaluation of Pain during Electrocutaneous Stimulation using a Log-Linearized Peripheral Arterial Viscoelastic Model

**DOI:** 10.1038/s41598-018-21223-1

**Published:** 2018-02-15

**Authors:** Hiroki Matsubara, Hiroki Hirano, Harutoyo Hirano, Zu Soh, Ryuji Nakamura, Noboru Saeki, Masashi Kawamoto, Masao Yoshizumi, Atsuo Yoshino, Takafumi Sasaoka, Shigeto Yamawaki, Toshio Tsuji

**Affiliations:** 1 Department of System Cybernetics, Graduate School of Engineering, Hiroshima University, 1-4-1 Kagamiyama, Higashi-Hiroshima, Hiroshima 739-8527 Japan; 2 College of Engineering, Academic Institute, Shizuoka University, 3-5-1, Johoku, Naka-ku, Hamamatsu, Shizuoka 432-8561 Japan; 3 Department of Anesthesiology and Critical Care, Graduate School of Biomedical and Health Sciences, Hiroshima University, 1-2-3 Kasumi, Minami-ku, Hiroshima, Hiroshima 734-8551 Japan; 4 Department of Cardiovascular Physiology and Medicine, Graduate School of Biomedical and Health Sciences, Hiroshima University, 1-2-3 Kasumi, Minami-ku, Hiroshima, Hiroshima 734-8551 Japan; 5 Department of Psychiatry and Neurosciences, Division of Frontier Medical Sciences, Graduate School of Biomedical and Health Sciences, Hiroshima University, 1-2-3 Kasumi, Minami-ku, Hiroshima, Hiroshima 734-8551 Japan; 6 Center of Innovation, KANSEI Innovation Program, Hiroshima University, 1-2-3 Kasumi, Minami-ku, Hiroshima, Hiroshima 734-8551 Japan

## Abstract

In clinical practice, subjective pain evaluations, e.g., the visual analogue scale and the numeric rating scale, are generally employed, but these are limited in terms of their ability to detect inaccurate reports, and are unsuitable for use in anesthetized patients or those with dementia. We focused on the peripheral sympathetic nerve activity that responds to pain, and propose a method for evaluating pain sensation, including intensity, sharpness, and dullness, using the arterial stiffness index. In the experiment, electrocardiogram, blood pressure, and photoplethysmograms were obtained, and an arterial viscoelastic model was applied to estimate arterial stiffness. The relationships among the stiffness index, self-reported pain sensation, and electrocutaneous stimuli were examined and modelled. The relationship between the stiffness index and pain sensation could be modelled using a sigmoid function with high determination coefficients, where *R*^2^ ≥ 0.88, *p* < 0.01 for intensity, *R*^2^ ≥ 0.89, *p* < 0.01 for sharpness, and *R*^2^ ≥ 0.84, *p* < 0.01 for dullness when the stimuli could appropriately evoke dull pain.

## Introduction

Pain plays a role in informing about potential damage to our body, and development of this perception is crucial for survival^1^. Three steps are involved in the process of pain perception. First, nerve endings convert painful stimulation applied to the skin to nerve signals, and transmit it to the dorsal horn of the spinal cord. Next, the signal ascends in the lateral spinothalamic tract to nuclei in the thalamus. Finally, the information reaches the somatosensory cortex, after which pain is perceived.

Generally, the following subjective pain evaluations are currently employed in clinical practice: medical interviews are conducted to acquire verbal expression of pain; the visual analogue scale (VAS); and the numeric rating scale (NRS)^2^. The VAS is a common pain intensity metric based on a 100-mm graduated line marked “No pain” on the far left and “Worst pain imaginable” on the far right; this metric involves measurement of the length from the no-pain point to the point indicated by the patient as representing the subjective pain intensity. The NRS involves the use of numerical values to represent pain intensity; it is scored on an 11-point range, with 0 representing no pain and 10 representing the worst pain imaginable^2–4^. In addition, Shimazu *et al*. proposed a system called Pain Vision for quantitative evaluation of pain. This system quantifies the pain intensity using electric current by comparing the sensory quantities induced by pain and that induced by a perceptible, but not painful, stimulus current^5^. However, subjective evaluation methods have limitations in detecting inaccurate reports and are unsuitable for patients under general anaesthesia or those with dementia.

To solve this problem, several objective and quantitative evaluation methods have been proposed. For example, Imasato *et al*.^6^ have reported that measurement of immune substance P in cerebrospinal fluid can be used for an objective, if invasive, evaluation of pain intensity in patients with spinal diseases. In terms of non-invasive evaluations, responses of autonomic nerve activity to pain have been measured. Fukushima *et al*. reported that the power spectral density of heartbeats between 0.04 and 0.4 Hz, as determined by heart rate analysis^7^, can be used to evaluate pain and discomfort during dental treatment^8^. It is also well-known that peripheral arteries respond rapidly to regulation by the sympathetic nervous system. Moreover, Kohnen *et al*. have reported that the photoplethysmogram amplitude is reduced when the participant experiences pain^9^. However, Nakamura *et al*. have reported that this amplitude may be unsuitable for the evaluation of autonomic nerve activity, as photoplethysmograms are affected by blood pressure changes in the absence of autonomic nerve activation^10^. Our research group therefore modelled the dynamic characteristics of the peripheral arterial wall, using a linear mechanical impedance model based on measured photoplethysmograms (PPGs) and blood pressure variation, and proposed a method for estimating peripheral sympathetic nerve activity using this model^11^. We also evaluated pain intensity during mechanical stimulation by using the arterial impedance model and analysed the relationship between pain intensity and the dynamic characteristics of the peripheral arterial wall^12^. However, the non-linear characteristics of arterial stiffness were not taken into consideration, which can be problematic, as blood pressure variations affect evaluation indices derived from the model^13^. In addition, the qualitative aspects of pain, such as dullness and sharpness^14^, have not yet been quantified.

This paper thus proposes a system for evaluating pain using a log-linearized peripheral arterial viscoelastic model, which considers the nonlinear effect of blood pressure variations. Using the proposed system, we examined the relationships between the sensation of pain, including its intensity and qualities, electrocutaneous stimuli, and the dynamic characteristics of the peripheral arterial wall controlled by the sympathetic nervous system.

## Methods

### Log-linearized peripheral arterial viscoelastic model

Previously, our group proposed a log-linearized peripheral arterial viscoelastic model that considers the nonlinear relationship between arterial wall impedance and blood pressure^13^. The model allowed estimation of arterial viscoelasticity by eliminating the effects caused by blood pressure variation that are unrelated to sympathetic nerve activity, and can thus quantitatively evaluate sympathetic nerve activity. The model is given by the following equation:

1 $${P}_{b}(t)=\tilde{\mu }\ddot{\varepsilon }(t)+\tilde{\eta }\dot{\varepsilon }(t)+\exp \{\tilde{\beta }\varepsilon (t)+{P}_{{\tilde{\beta }}_{0}}+{P}_{{\tilde{\beta }}_{nl}}(\varepsilon (t))\},$$

where *t* represents time, $\tilde{\mu }$, $\tilde{\eta }$ and $\tilde{\beta }$ are the inertia, viscosity, and stiffness of the arterial wall, respectively. *P*_*b*_(*t*), *ε*(*t*), $\dot{\varepsilon }(t)$, and $\ddot{\varepsilon }(t)$ represent the blood pressure, the strain of the arterial diameter, the strain velocity, and the strain acceleration, respectively. ${P}_{{\tilde{\beta }}_{0}}$ is the standard blood pressure, whereas the non-linear term ${P}_{{\tilde{\beta }}_{nl}}(\varepsilon (t))$ is a stiffness pressure component originating in the vein.

For simplicity, assuming that the strain *ε* is in proportion to the photoplethysmogram *P*_*l*_(*t*), Equation (1) can thus be rewritten as follows, by replacing the symbols:

2 $${P}_{b}(t)=\mu {\ddot{P}}_{l}(t)+\eta {\dot{P}}_{l}(t)+\exp \{\beta {P}_{l}(t)+{P}_{b{\beta }_{0}}+{P}_{b{\beta }_{nl}}({P}_{l}(t))\},$$

where, ${P}_{b{\beta }_{0}}$ corresponds to the standard blood pressure, and ${P}_{b\beta nl}(r(t))$ corresponds to the pressure originating in the vein. Please see Appendix for details regarding formulation of the model.

### Two-step procedure for estimating viscoelasticity parameters

The viscoelasticity parameters *μ*, *η* and *β*_*A*_ for each heartbeat are estimated by a two-step procedure^13^.

In the first step, the stiffness blood pressure in Equation (2) is approximated by a linear approximate equation using a Maclaurin series expansion, based on the presumption that the higher-order terms (exceeding the second-order term) are sufficiently small, as follows:

3 $${P}_{b}(t)\approx \mu {\ddot{P}}_{l}(t)+\eta {\dot{P}}_{l}(t)+\exp \{{P}_{b{\beta }_{0}}+{P}_{b{\beta }_{nl}}\mathrm{(0)}\}+{\beta }_{A}{P}_{l}(t).$$

In this equation, *β*_*A*_ is defined as follows:

$${\beta }_{A}={C}_{1}\,\exp \{{P}_{b{\beta }_{0}}+{P}_{b{\beta }_{nl}}\mathrm{(0)}\}{C}_{1}={\beta +\frac{d{P}_{b{\beta }_{nl}}({P}_{l}(t))}{d{P}_{l}(t)}|}_{{P}_{l}(t)=0},$$

where time *t*_0_ is defined as an arbitrary reference time in the cardiac cycle, such as R wave timing. The viscoelasticity parameters of the artery at the arbitrary time *t* can be expressed using the following equation:

4 $$\begin{array}{rcl}d{P}_{b}(t) & = & \mu d{\ddot{P}}_{l}(t)+\eta d{\dot{P}}_{l}(t)+{\beta }_{A}d{P}_{l}(t),\\ d{P}_{b}(t) & = & {P}_{b}(t)-{P}_{b}({t}_{0}),\\ d{\ddot{P}}_{l}(t) & = & {\ddot{P}}_{l}(t)-{\ddot{P}}_{l}({t}_{0}),\\ d{\dot{P}}_{l}(t) & = & {\dot{P}}_{l}(t)-{\dot{P}}_{l}({t}_{0}),\\ {P}_{l}(t) & = & {P}_{l}(t)-{P}_{l}({t}_{0}\mathrm{).}\end{array}$$

*μ*, *η* and *β*_*A*_ are then estimated by implementing the least-squares method on the data for each heartbeat, using Equation (5) where *β*_*A*_ is an approximation of the stiffness characteristic.

In the second step, substituting *μ* and *η* into Equation (2) yields an equation that can be used to separate the stiffness blood pressure terms from other terms, and the following can be obtained by taking the exponent on both sides of the equation:

5 $$\beta {P}_{l}(t)+{P}_{b{\beta }_{0}}+{P}_{b{\beta }_{nl}}({P}_{l}(t))=\,\mathrm{ln}\,\{{P}_{b}(t)-\mu {\ddot{P}}_{l}(t)-\eta {\dot{P}}_{l}(t)\}.$$

In the same manner as in Equation (5), the difference of Equation (5) at arbitrary time *t* and the reference time *t*_0_ of the cardiac cycle gives the following equation:

6 $$\beta \,d\,{P}_{l}(t)+{P}_{b{\beta }_{nl}}({P}_{l}(t))-{P}_{b{\beta }_{nl}}({P}_{l}({t}_{0}))=\,\mathrm{ln}\{\tfrac{{P}_{b}(t)-\mu {\ddot{P}}_{l}(t)-\eta {\dot{P}}_{l}(t)}{{P}_{b}({t}_{0})-\mu {\ddot{P}}_{l}({t}_{0})-\eta {\dot{P}}_{l}({t}_{0})}\}\mathrm{.}$$

As ${P}_{b{\beta }_{nl}}({P}_{l}(t))-{P}_{b{\beta }_{nl}}({P}_{l}({t}_{0}))$ equals 0, the stiffness index *β* can then be estimated for each heartbeat using the least-squares method. Please note that Equation (6) is applicable for estimating the stiffness index *β* only when the log-linearized stiffness term and strain of the arterial diameter are linearly related. When the blood pressure *P*_*b*_(*t*) falls below the mean blood pressure, making the artery significantly stiff, the above condition is not fulfilled. The stiffness index *β* is therefore estimated using the data of the area above the mean blood pressure.

As it has been reported that the arterial stiffness index *β* responds sensitively to direct stimulation of sympathetic nerves^13^, the arterial stiffness index *β* has been used as an evaluation index for estimating pain-evoked changes in sympathetic nerve activity.

### Measurement system

**Figure 1:**
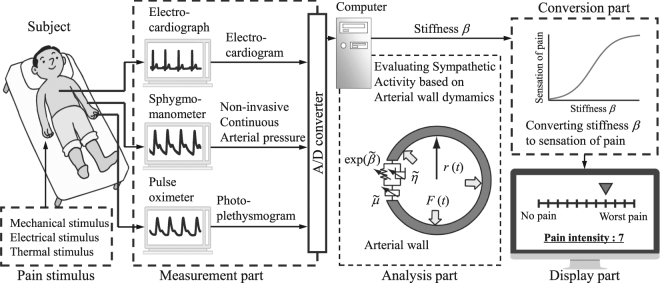
Overview of the system for evaluating pain intensity, dullness, and sharpness.

Figure 1 shows the proposed system for evaluating the sensation of pain. The system consists of four parts: the measurement, analysis, conversion, and display parts.

The measurement part simultaneously measures the electrocardiogram, non-invasive continuous blood pressure *P*_*b*_(*t*), and photoplethysmogram *P*_*l*_(*t*). Electrocardiogram signals were measured with a three-lead electrocardiograph, and non-invasive left radial arterial blood pressure was measured using a biological information monitor (BP-608 Evolution II CS, Omron Colin, Tokyo, Japan). Photoplethysmograms were measured using a pulse oximeter (OLV-3100, Nihon Kohden Corp., Tokyo, Japan). These data were stored at 1000 Hz on a computer using an analog/digital converter (CSI-360116, Interface Corp., Hiroshima, Japan).

The analysis part estimates the arterial stiffness index *β* using the method described in the previous section, as it responds sensitively to direct stimuli on the sympathetic nerve^13^. To assure the estimation accuracy, the determination coefficient *R*^2^ between the measured blood pressure values and the estimated blood pressure values is calculated, and evaluation was performed only when *R*^2^ ≥ 0.9.

In the conversion part, the estimated stiffness index *β* is converted to an NRS value, which represents pain sensation of the average person. As the NRS is restricted to a range of 0 to 10, the conversion equation is defined as a sigmoid function, as follows:

7 $${\rm{e}}{\rm{N}}{\rm{R}}{\rm{S}}=\{\begin{array}{cc}{\textstyle \tfrac{20}{1+\exp (a{({\beta }_{{\rm{n}}}-1)}^{-b})}} & ({\beta }_{{\rm{n}}} > 1)\\ 0 & ({\beta }_{{\rm{n}}}\le 1)\end{array},$$

where 0 < eNRS < 10 represents NRS converted from *β*, *a* and *b* are the parameters that are experimentally determined by the relationship between the sensation of pain and arterial stiffness index *β*. *β*_n_ is calculated by normalizing the maximum value of *β*, when no stimulation was applied; thus, it represents the change rate in *β*.

In the display part, the evaluated values of pain sensation converted from *β* are shown for examination.

The datasets generated and/or analysed during the current study are available from the corresponding author on reasonable request.

### Experimental configurations

**Figure 2:**
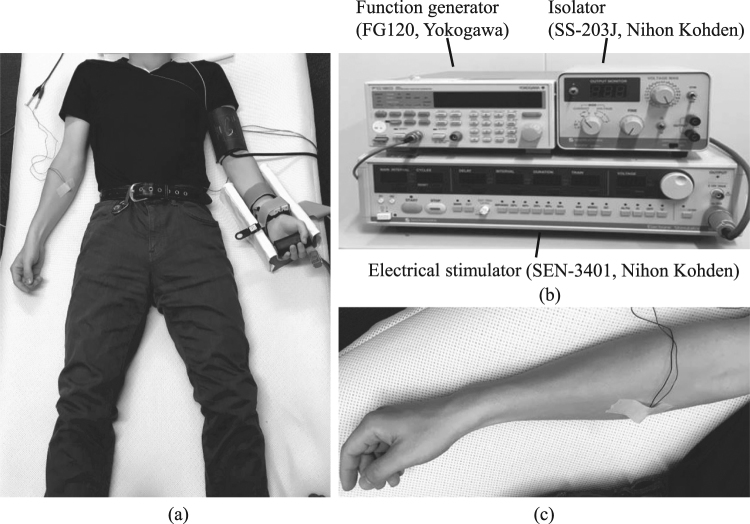
Experimental environment: (**a**) a participant during measurement, (**b**) electrocutaneous stimulation apparatus, (**c**) electrocutaneous stimulation site.

Experiments were conducted in accordance with the Declaration of Helsinki. Informed consent was obtained from all study participants before the experiments were performed, and the study was approved by the Hiroshima University Ethics Committee (Registration number: E-17). Informed consent for publication of identifying information/images in an online open-access publication was obtained from the subject in Fig. 2.

To verify the ability of the proposed system to evaluate the pain sensation, including the pain intensity, sharpness (from fast pain), and dullness (from slow pain), electrocutaneous stimuli were applied to the participants by altering current amplitude and frequency. Eleven healthy male participants (mean age ± S.D.: 22.8 ± 1.7 yrs.) participated in this experiment.

Figure 2 shows the experimental environment. Each participant was placed in a supine position with electrocardiograph sensors attached to the chest, a blood pressure monitor was attached to the left upper arm, and a photoplethysmogram sensor was attached to the index finger. To reduce the biological reactions other than pain, the participant wore a blindfold and noise-cancelling headphones (ATH-ANC9, Audio-technica, Tokyo, Japan).

In the experiment, sine-wave electrocutaneous stimuli *I*(*t*) of 5 Hz, 125 Hz, and 250 Hz were applied to participants. Here, the 250-Hz and 5-Hz stimuli were respectively used to stimulate C fibres to generate sharp pain and A*δ* fibres to generate dull pain, and the 125-Hz stimulus was intended to generate both sharp and dull pain. The electrocutaneous stimulation current was produced by an electrical stimulator (SEN-3401, Nihon Kohden Corp., Tokyo, Japan), an isolator (SS-203J, Nihon Kohden Corp., Tokyo, Japan), and a function generator (FG120, Yokogawa Electric Corp., Tokyo, Japan). The stimulating current was applied to the skin surface on the inner side of the right forearm through an electrode (NM-990W, Nihon Kohden Corp., Tokyo, Japan) (Fig. 2(b,c)).

Since the pain sensation differed substantially between individuals, a standard current amplitude was adjusted for each participant prior to this experiment. That is, the amplitude at which the participant reported the degree of pain as ‘3’ on the NRS was set as the standard amplitude.

An experimental block consists of seven sequential trials. Each trial starts with 20 s of rest, followed by a 24-s task of continuous electrocutaneous stimulation and 20 s of pain evaluation, so that the duration of the experimental block was 448 s. The stimulus conditions in the sequential tasks were varied by applying currents of different amplitude in the order of 1.5 times, 1.0 times, 0.5 times, 0 times, 0.5 times, 1.0 times, and 1.5 times as large as the standard current amplitude. This condition was configured to reduce the influence of the participants’ anxiety during the trials by experiencing the most intense pain at the first trial. In the 20 s of pain evaluation, the participants verbally reported pain intensity, sharpness, and dullness by using the NRS.

### Data analysis

The values of *β*_n_ were compared between when no stimulation and when 1.5 times the standard stimulation were respectively applied to the participant, in order to quantify the effect of the stimulation on the stiffness index. Tukey’s test was used to determine the significance of differences, with the significance level set at *p* < 0.05.

The mean values and standard deviation of the self-reported pain intensities for the respective stimulus levels were then calculated from data obtained from all participants to evaluate relationships among electrocutaneous stimuli, *β*_n_, and self-reported pain intensity. The relationships between electrocutaneous stimuli and *β*_n_ were modelled using a first-order linear function. The following sigmoid function was used to model the relationship between self-reported pain intensity and electrocutaneous stimulus level.

8 $$e{\beta }_{{\rm{n}}}=\frac{10}{1+\exp (a{S}^{-b})}+1,$$

where *eβ*_n_ is the pain intensity predicted by the model, and *S* = 0, 0.5, 1, 1.5 represents the stimulus level.

The frequencies of electrocutaneous stimuli and the mean value of self-reported pain sensation of all the participants were also compared. The reported NRS values of pain intensity, sharpness, and dullness for stimulation at 1.5 times as large as the standard current amplitude were used to test capability of *β*_n_ on detecting difference in self-reported pain sensation, because the stimulus was intended to raise the strongest sensation. Tukey’s test was used to determine the significance of differences, with the significance level set at *p* < 0.05. To investigate the relationships between *β*_n_ and subjective pain sensation (sharpness and dullness), the sigmoid function (7) was used.

## Results

**Figure 3:**
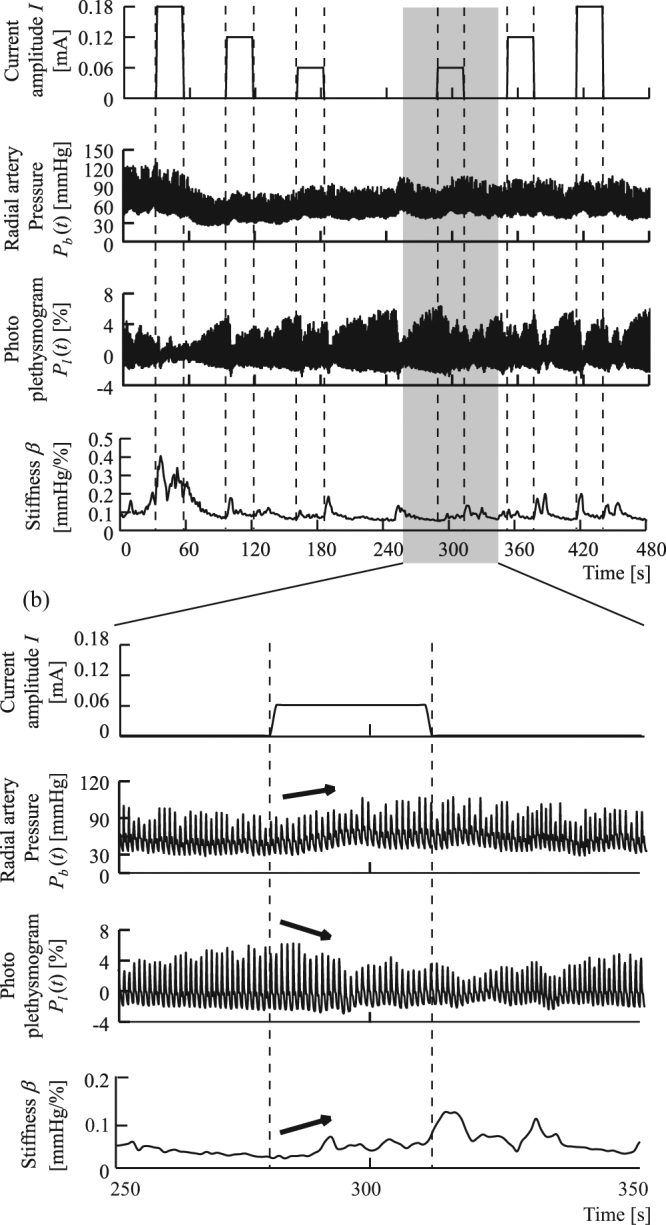
Measured radial arterial pressure and photoplethysmogram and estimated stiffness index *β* from Sub. A when 125-Hz electrocutaneous stimuli were applied.

Figure 3 shows the measured signals and analysed signals of participant A during the application of electrocutaneous stimulation at 125 [Hz]. From top to bottom, each figure shows the maximum current amplitude of electrocutaneous stimulation *I*(*t*), non-invasive blood pressure *P*_*b*_(*t*), photoplethysmograms *P*_*l*_(*t*), and stiffness index *β*. Figure 3 indicates that the stimulus increased blood pressure and decreased photoplethysmogram amplitude. The stiffness index *β* also changed depending on the electrocutaneous stimulation amplitude.

**Figure 4:**
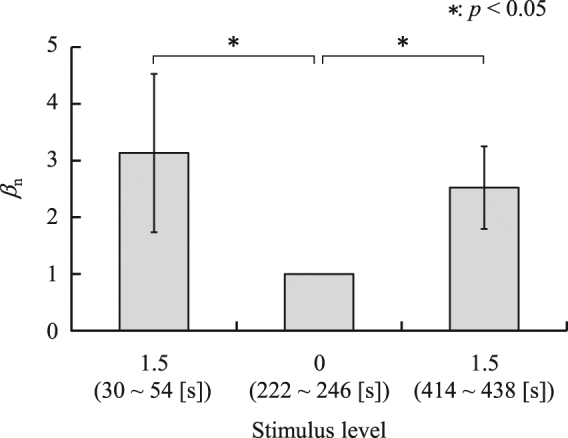
Comparison of *β*_n_ when 125-Hz electrocutaneous stimuli were applied. In the period from 30 s to 54 s, and that from 414 s to 438 s, a stimulus 1.5 times as large as the standard stimulus was applied to the participants. In the period from 222 s to 246 s, no stimulation was applied.

To quantify responses of the stiffness index *β* to the stimulation, the values of *β*_n_ were compared between no stimulation (222–246 seconds) and 1.5 times the standard stimulation (30–54 s and 414–438 s). Figure 4 shows *β*_n_ averaged over all participants. The figure shows that *β*_n_ significantly differed (*p* < 0.05) between the no-stimulation and 1.5 times the standard stimulation conditions.

**Figure 5:**
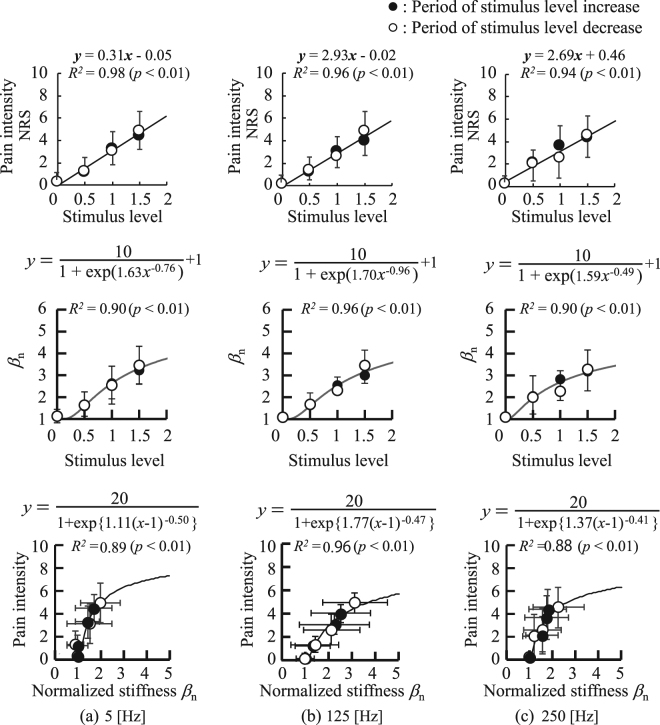
Relationship between the estimated *β*_n_ and the applied stimulus level, self-reported pain intensity, and the applied stimulus level, and the self-reported pain intensity and estimated *β*_n_: (**a**) 5 Hz, (**b**) 125 Hz, (**c**) 250 Hz.

Figure 5 shows the relationships between the stimulation levels, *β*_n_ and the self-reported pain intensity averaged over all participants for the respective stimulus levels. From top to bottom, the figures show the relationship between stimulus level and self-reported pain intensity, stimulus level and *β*_n_, and *β*_n_ and self-reported pain intensity. Significant coefficients of determination between the stimulus level and self-reported pain intensity were found: 5 Hz: *R*^2^ = 0.94, *p* < 0.01, 125 Hz: *R*^2^ = 0.98, *p* < 0.01, and 250 Hz: *R*^2^ = 0.92, *p* < 0.01. The relationships between the stimulation level and *β*_n_ for each stimulation frequency were modelled using Equation 8, which yielded high coefficients of determination: 5 Hz: *R*^2^ = 0.90, *p* < 0.01, 125 Hz: *R*^2^ = 0.96, *p* < 0.01, and 250 Hz: *R*^2^ = 0.90, *p* < 0.01. The relationships between *β*_n_ and self-reported pain intensity for each stimulation frequency were modelled using Equation 7, which yielded high coefficients of determination: 5 Hz: *R*^2^ = 0.89, *p* < 0.01, 125 Hz: *R*^2^ = 0.96, *p* < 0.01, and 250 Hz: *R*^2^ = 0.88, *p* < 0.01.

**Figure 6:**
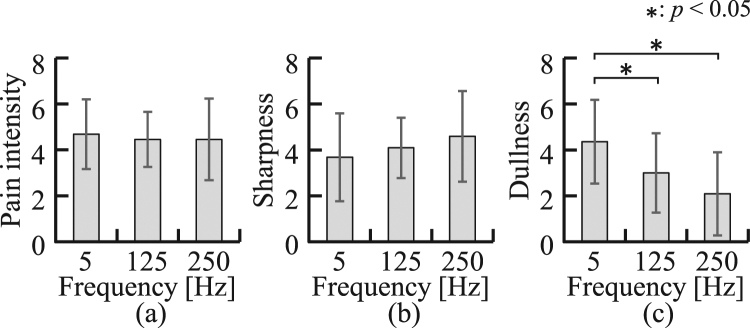
Comparison of self-reported NRS values of pain sensation when a stimulation current 1.5 times as large as the standard stimulation current was applied: (**a**) pain intensity, (**b**) sharpness, (**c**) dullness.

In addition, the relationships between *β*_n_ and self-reported pain sensations, including sharpness and dullness are evaluated. Figure 6 compares the mean self-reported pain sensation of all participants to whom a stimulus 1.5 times the standard stimulus was applied for different stimulus frequencies. The figure indicates that there were no significant differences between the pain intensity and sharpness for any stimulus frequency, but significant differences were found in terms of dullness between a stimulation frequency of 5 Hz and those of both 125 Hz and 250 Hz.

**Figure 7:**
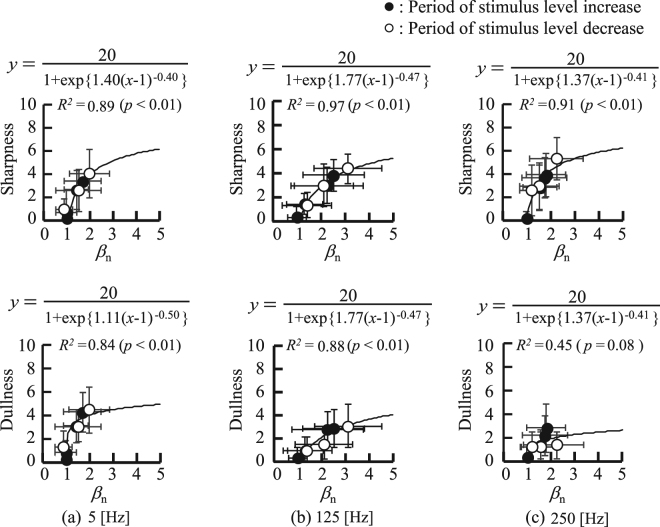
Relationships between the *β*_n_ and self-reported pain sensation, including sharpness and dullness: (**a**) 5 Hz, (**b**) 125 Hz, (**c**) 250 Hz.

Figure 7 shows the relationships between *β*_n_ and self-reported dullness and sharpness on the NRS averaged over all participants for the respective stimulus levels. From top to bottom, the figures show the relationships between *β*_n_ and sharpness, and *β*_n_ and dullness, with respect to stimulus frequency: respectively, (a) 5 Hz, (b) 125 Hz, and (c) 250 Hz. The relationships between *β*_n_ and sharpness for each stimulation frequency were modelled using Equation 7, which yielded high coefficients of determination: 5 Hz: *R*^2^ = 0.89, *p* < 0.01, 125 Hz: *R*^2^ = 0.97, *p* < 0.01, and 250 Hz: *R*^2^ = 0.91, *p* < 0.01. The relationships between *β*_n_ and dullness were also modelled using Equation 7 and yielded high coefficients of determination for stimulus frequencies of 5 Hz and 125 Hz, whereas no significant coefficient of determination was found for the stimulus frequency at 250 Hz; 5 Hz: *R*^2^ = 0.84, *p* < 0.01, 125 Hz: *R*^2^ = 0.88, *p* < 0.01, and 250 Hz: *R*^2^ = 0.45, *p* = 0.08.

## Discussion

In an attempt to establish an objective pain evaluation method, this paper proposed a system for evaluating the pain sensation evoked by electrocutaneous stimuli using *β*_n_. *β*_n_ is derived from a log-linearized peripheral arterial viscoelastic model that considers the non-linear relationship between the arterial stiffness and blood pressure. We then examined relationships between electrocutaneous stimulation, self-reported pain sensation, and *β*_n_.

First, we confirmed that electrocutaneous stimuli increased blood pressure and decreased plethysmogram amplitude as shown in Fig. 3. This result was consistent with a previous study that reported that the peripheral arterial wall contracts in response to sympathetic nerve activity^15^. Because participants wore a blindfold and a noise-reduction headphone, respectively, to reduce visual and auditory effects on sympathetic nerve activity, electrocutaneous stimulation was the main source of the responses.

The relationships between *β*_n_ and pain sensation were then examined from the following perspectives: pain detection ability, capacity for self-reported pain intensity evaluation, and finally, the capacity for pain sensation evaluation, including dullness and sharpness.

It was confirmed that *β*_n_ can detect pain stimuli, because a significant difference in *β*_n_ was found between conditions of no-stimulation and the maximum stimulation applied in the experiments (see Fig. 4). High coefficients of determination confirmed between the self-reported pain intensity and the stimulus level indicate that the pain intensity linearly increases with stimulus level in this experimental configuration. On the other hand, we found a sigmoidal relationship between the stimulus level and *β*_n_ as well as *β*_n_ and self-reported pain intensity (see Fig. 5) suggesting that the saturation effect has to be taken into account when evaluating pain using *β*_n_. The high coefficient of determination yielded by Equation (7) demonstrates the effectiveness of using *β*_n_ for evaluating pain intensity.

The stimulus frequency dependence of the self-reported pain sensation was also examined. We found that the self-reported pain intensity depends only on the stimulus level, but not on the stimulus frequency (see Fig. 6(a)). Although there was a tendency for the stimulus frequency to increase with self-reported sharpness (see Fig. 6(b)), the fact that no significant difference was found in self-reported sharpness between different stimulus frequencies indicates that all stimulus frequencies evoked fast pain, accompanied with sharpness^14^. On the other hand, the stimulation frequency decreased self-reported dullness (see Fig. 6(c)). This is consistent with a previous study reporting that the electrical stimulation at 5 Hz primarily stimulates C-fibres, which transfer dull pain^16^. This result also indicates that dullness does not completely depend on pain intensity.

Finally, the ability of *β*_n_ to evaluate pain sensation was tested. The test results demonstrated that sharpness reported by the average participant can be evaluated by applying the sigmoid model described by Equation (7) on *β*_n_, as high determination coefficients were confirmed for all stimulus frequencies (see Fig. 7). On the other hand, *β*_n_ could not evaluate dullness for stimulus frequencies at 250 Hz. This is because the stimulus at 250 Hz did not cause dullness as much as did the other frequencies. However, the results also demonstrate that *β*_n_ can evaluate dullness evoked by stimulation at 5 Hz and 125 Hz, which can sufficiently evoke dullness.

The experimental results indicate that the proposed method could evaluate pain intensity, thereby allowing the system to contribute to medical assessment of patients who cannot report pain sensation. Evaluating pain before and after surgery, or before and after taking analgesic medication, is important to control the state of the patient in clinical practice. The proposed system enables quantitative evaluation without interviewing the patient or obtaining a subjective evaluation of pain. This is an advantage of the proposed system, which cannot be achieved by the current subjective evaluation using VAS, NRS^2–4^, or quantitative evaluation using Pain Vision^5^, which requires an interview. In addition, there are cases where patients report false information for their personal gain. The proposed system can add important information to assess the pain condition of the patient, thereby supporting accurate diagnosis. Please note that the individual differences in pain sensation may limit adaptation of the model to a specific individual. The effect of intra- and inter-individual differences on the relationship between each pair of stimulus levels, *β*_n_, as well as on self-reported pain, must be clarified in the near future.

In conclusion, this study revealed reliable relationships between each pair of *β*_n_, the electrocutaneous stimulus level, and self-reported pain intensity. To assure the effectiveness of the proposed pain evaluation method, a further study is required to endorse the relationship between *β*_n_ and pain recognition. We thus plan to compare *β*_n_ with brain activities by performing functional magnetic resonance imaging experiments.

## Electronic supplementary material

Appendix
