# Supplementary material for: Quantitative Evaluation of Pain during Electrocutaneous Stimulation using a Log-Linearized Peripheral Arterial Viscoelastic Model
Source: Sci Rep. 2018 Feb 15;8:3091. doi: 10.1038/s41598-018-21223-1 (PMC5814565; doi:10.1038/s41598-018-21223-1)
Supplement: Supplementary file 1 — Appendix [file 41598_2018_21223_MOESM1_ESM.pdf]

Appendix

Quantitative Evaluation of Pain during Electrocutaneous Stimulation using a Log-Linearized Peripheral Arterial Viscoelastic Model

Hiroki Matsubara[1], Hiroki Hirano[1], Harutoyo Hirano[2], Zu Soh[1], Ryuji Nakamura[3], Noboru Saeki [3], Masashi Kawamoto[3], Masao Yoshizumi[4], Atsuo Yoshino[5], Takafumi Sasaoka[6], Shigeto Yamawaki[5, 6], and Toshio Tsuji[1,*]

1 Department of System Cybernetics, Graduate School of Engineering, Hiroshima University, 1-4-1 Kagamiyama, Higashi-Hiroshima, Hiroshima 739-8527, Japan
2 College of Engineering, Academic Institute, Shizuoka University, 3-5-1, Johoku, Naka-ku, Hamamatsu, Shizuoka, 432-8561, Japan
3 Department of Anesthesiology and Critical Care, Graduate School of Biomedical and Health Sciences, Hiroshima University, 1-2-3 Kasumi, Minami-ku, Hiroshima, Hiroshima, 734-8551, Japan
4 Department of Cardiovascular Physiology and Medicine, Graduate School of Biomedical and Health Sciences, Hiroshima University, 1-2-3 Kasumi, Minami-ku, Hiroshima, Hiroshima, 734-8553, Japan
5 Department of Psychiatry and Neurosciences, Division of Frontier Medical Sciences, Graduate School of Biomedical and Health Sciences, Hiroshima University, 1-2-3 Kasumi, Minami-ku, Hiroshima, Hiroshima, 734-8551, Japan
6 Center of Innovation, KANSEI Innovation Program, Hiroshima University, 1-2-3 Kasumi, Minami-ku, Hiroshima, Hiroshima, 734-8551, Japan
* tsuji@bsys.hiroshima-u.ac.jp

A LOG-LINEARIZED PERIPHERAL ARTERIAL VISCOELASTIC INDEX

In quantitative evaluation of the mechanical characteristics of peripheral arteries, the following three points should be considered: (i) arterial wall mechanical characteristics can be quantitatively evaluated using mechanical impedance parameters, such as stiffness and viscosity; (ii) stiffness changes caused by sympathetic nerve activity can be estimated, considering nonlinearity between intravascular pressure and arterial diameter; and (iii) specific features of peripheral arteries (e.g., effects of veins and accumulation in arterioles) can be expressed. This section introduces a mechanical peripheral arterial wall model that was designed in consideration of these three points.

Figure 1 shows the proposed mechanical impedance model of the peripheral arterial wall[1,2]. The arterial wall's characteristics in an arbitrary radial direction are considered, and are shown with changes in blood pressure and strain of the arterial diameter, as follows:

$$\begin{aligned} P_b(t) &= P_\mu(t) + P_\eta(t) + P_\beta(t) \\ &\approx \tilde{\mu}\ddot{\epsilon}(t) + \tilde{\eta}\dot{\epsilon}(t) + P_\beta\big(\epsilon(t)\big). \end{aligned} \tag{1}$$

$P_b(t)$ is the blood pressure at time t; $P_\mu(t)$, $P_\eta(t)$ and $P_\beta(t)$ are the pressures originating from inertia, viscosity, and stiffness, respectively; and $\tilde{\mu}$ and $\tilde{\eta}$ are the arterial wall inertia and viscosity, respectively. The parameters $\epsilon(t)$, $\dot{\epsilon}(t)$, and $\ddot{\epsilon}(t)$ represent the strain of the arterial diameter, strain velocity, and strain acceleration, respectively. Here, we assume that $\tilde{\mu}\ddot{\epsilon}(t)$ and $\tilde{\eta}\dot{\epsilon}(t)$ include an intervascular pressure component that originated from the vein. In addition, considering the nonlinearity between intravascular pressure and the strain, the pressure originating from vein stiffness is expressed as $P_{\tilde{\beta}}\left(\epsilon(t)\right)$. This assumption is verified by the experimental results, as described in a later section.

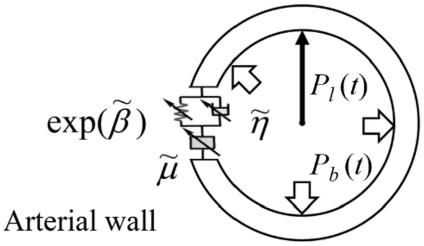

Fig. 1 Mechanical impedance model of a peripheral arterial wall.

$P_{\tilde{\beta}}\left(\epsilon(t)\right)$ is then formulated based on the previous studies and can be viewed as the extended version of the arterial impedance model proposed by Simon *et al*. based on the Voigt model[3,4] through addition of the inertial term and including consideration of the vein effect. The relationship between blood pressure and arterial diameter has been extensively investigated in previous experiments. Nagasawa *et al*. and Hayashi *et al*. measured canine intravascular pressure and the diameter of the arterial wall, using a femoral artery on an *in vitro* basis to support examination of the artery's mechanical properties[5,6]. They found that the canine femoral artery exhibits distensibility in the range of 60–180 mmHg, and that the ratio of the logarithm of intravascular pressure to standard pressure is proportional to the ratio of the arterial wall diameter to its diameter with standard intravascular pressure. However, in the case of low blood pressure, the artery stiffens because the variation in blood vessel diameter, which accompanies increased intravascular pressure, becomes small[5]. Because of this fact, we assumed that human peripheral arterial characteristics are also nonlinear[1,2], as observed in the canine femoral artery, in terms of arterial wall mechanical characteristics. The logarithm pressure originating from arterial stiffness, considering accumulation in arterioles in the peripheral area, is expressed as follows:

$$\ln P_{\tilde{\beta}}\left(\epsilon(t)\right) = \tilde{\beta}\left(\epsilon(t)\right) + P_{\tilde{\beta}_0} + P_{\tilde{\beta}_{nl}}\left(\epsilon(t)\right). \quad (2)$$

$\tilde{\beta}$ is the arterial stiffness relating to logarithm pressure, and $P_{\tilde{\beta}_0}$ is the constant pressure acting on the arterial wall. $P_{\tilde{\beta}_{nl}}\left(r(t)\right)$ is a pressure originating from vein stiffness that cannot be log-linearized and accrues when intravascular pressure falls below a certain threshold. Equation (2) can be substituted into Equation (1) after taking the exponent on both sides of Equation (2), and arterial impedance properties can be expressed as follows:

$$P_b(t) \approx \tilde{\mu}\,\ddot{\epsilon}(t) + \tilde{\eta}\,\dot{\epsilon}(t) + \exp\left\{\tilde{\beta}\epsilon(t) + P_{\tilde{\beta}_0} + P_{\tilde{\beta}_{nl}}\left(\epsilon(t)\right)\right\}. \quad (3)$$

Because it is difficult to measure the strain $\epsilon(t)$ directly *in vivo*, the strain is approximated using PPGs, which are measured with a pulse oximeter[7,8].

$$P_l(t) \cong k_p\epsilon(t) + P_{l_0}. \quad (4)$$

$P_l(t)$ is the PPG, k_p is a constant of proportion, and P_{l_0} is an offset constant. Equation (3) can be expressed using Equation (4), as follows:

$$P_b(t) = \mu\ddot{P}_l(t) + \eta\dot{P}_l(t) + \exp\left\{\beta P_l(t) + P_{b_{\beta_0}} + P_{b_{\beta_{nl}}}\left(P_l(t)\right)\right\}, \quad (5)$$

where

$$\mu = \frac{\tilde{\mu}}{k_p}, \eta = \frac{\tilde{\eta}}{k_p}, \beta = \frac{\tilde{\beta}}{k_p}, P_{b\beta_0} = F_{\tilde{\beta}_0} - \frac{\tilde{\beta} P_{l_0}}{k_p},$$

and $P_{b\beta_{nl}}(P_l(t)) = P_{\tilde{\beta}_{nl}}(\epsilon(t))$ represents blood pressure, which cannot be log-linearized and accrues when intravascular pressure decreases. The parameters μ, η, and β are the inertia, viscosity, and stiffness of the arterial wall, respectively. Equation (5) represents the log-linearized peripheral arterial viscoelastic model, which expresses peripheral arterial dynamic characteristics.

The method for estimating the impedance parameters inertia μ, viscosity η, and stiffness β, is outlined as follows. Estimating three impedance parameters at the same time is difficult because of the characteristic of $P_{b\beta_{nl}}(P_l(t))$, which cannot express linearity, even if $P_{b\beta_{nl}}(P_l(t))$ is used for the logarithm. Accordingly, estimation of parameters is performed in two stages.

In the first stage, the stiffness component of Equation (5) is approximated by the first-order term function by Maclaurin series expansion, based on the presumption that second- and higher-order terms are sufficiently small:

$$\exp\left\{\beta P_l(t) + P_{b\beta_0} + P_{b\beta_{nl}}(P_l(t))\right\} \approx \exp\left\{P_{b\beta_0} + P_{b\beta_{nl}}(0)\right\} + \beta_A P_l(t), \tag{6}$$

The following are details of Maclaurin series expansion: let

$$f(P(t)) = \exp\left\{\beta P_l(t) + P_{b\beta_0} + P_{b\beta_{nl}}(P_l(t))\right\}. \tag{7}$$

if the second- and higher-order terms are negligible, then

$$f(P(t)) \approx f(0) + \frac{df(0)}{dP_l(t)} P_l(t).$$

Since

$$f(0) = \exp\left\{P_{b\beta_0} + P_{b\beta_{nl}}(0)\right\},$$

$$\beta_A = \frac{df(0)}{dP_l(t)} = C_1 \exp\left\{P_{b\beta_0} + P_{b\beta_{nl}}(0)\right\},$$

$$C_1 = \beta + \left.\frac{dP_{b\beta_{nl}}(P_l(t))}{dP_l(t)}\right|_{P_l(t)=0}.$$

Therefore, Equation (5) can be approximated as follows:

$$P_b(t) \approx \mu \ddot{P}_l(t) + \eta \dot{P}_l(t) + \exp\left\{P_{b\beta_0} + P_{b\beta_{nl}}(0)\right\} + \beta_A P_l(t). \tag{8}$$

By using variations of the variables from the nominal values at time t_0, the dynamic characteristics of the artery can be expressed as follows:

$$dP_b(t) = \mu d\ddot{P}_l(t) + \eta d\dot{P}_l(t) + \beta_A dP_l(t), \tag{9}$$

where $dP_b(t) = P_b(t) - P_b(t_0)$, $d\ddot{P}_l(t) = \ddot{P}_l(t) - \ddot{P}_l(t_0)$, $d\dot{P}_l(t) = \dot{P}_l(t) - \dot{P}_l(t_0)$, and $dP_l(t) = P_l(t) - P_l(t_0)$. The parameters μ, η, and β_A are then estimated, using the least-squares method for each heartbeat, via Equation (9). β_A is an approximation of the stiffness characteristics.

In the second stage, substituting the estimated inertia $\hat{\mu}$ and viscosity $\hat{\eta}$ in the first stage into Equation (6) yields an equation that can be used to separate the stiffness component from other components, as follows:

$$\exp\left\{\beta P_l(t) + P_{b_{\beta_0}} + P_{b_{\beta_{nl}}}\big(P_l(t)\big)\right\} = P_b(t) - \hat{\mu}\ddot{P}_l(t) - \hat{\eta}\dot{P}_l(t). \quad (10)$$

Taking the exponent on both sides of Equation (10), the following equation can be obtained:

$$\beta P_l(t) + P_{b_{\beta_0}} + P_{b_{\beta_{nl}}}\big(P_l(t)\big) = \ln\left\{P_b(t) - \hat{\mu}\ddot{P}_l(t) - \hat{\eta}\dot{P}_l(t)\right\}. \quad (11)$$

By using variations of the variables from the nominal values at time t_0 in the same manner as in Equation (9), Equation (11) can be expressed as follows:

$$\beta dP_l(t) + P_{b_{\beta_{nl}}}\big(P_l(t)\big) - P_{b_{\beta_{nl}}}\big(P_l(t_0)\big) = \ln\left\{\frac{P_b(t) - \hat{\mu}\ddot{P}_l(t) - \hat{\eta}\dot{P}_l(t)}{P_b(t_0) - \hat{\mu}\ddot{P}_l(t_0) - \hat{\eta}\dot{P}_l(t_0)}\right\} \quad (12)$$

Estimation of the stiffness parameter β is limited to the area where $P_{b_{\beta_{nl}}}\big(P_l(t)\big) = P_{b_{\beta_{nl}}}\big(P_l(t_0)\big)$. When the arterial pressure $P_b(t)$ falls below a threshold pressure TH and the artery is considerably stiff, the above condition is not fulfilled. Therefore, the stiffness parameter β is estimated under the area $P_b(t)$ > TH. Equation (12) is then approximated as follows, because $P_{b_{\beta_{nl}}}\big(P_l(t)\big) - P_{b_{\beta_{nl}}}\big(P_l(t_0)\big)$ is equal to 0.

$$\beta dP_l(t)\big|_{P_b(t)>\mathrm{TH}} = \ln\left\{\frac{P_b(t) - \hat{\mu}\ddot{P}_l(t) - \hat{\eta}\dot{P}_l(t)}{P_b(t_0) - \hat{\mu}\ddot{P}_l(t_0) - \hat{\eta}\dot{P}_l(t_0)}\right\}\Bigg|_{P_b(t)>\mathrm{TH}}. \quad (13)$$

Because the stiffness parameter β in the left side of the above equation is the only unknown parameter in Equation (13), it can be estimated for each heartbeat, using the least-squares method, via Equation (13).

As mentioned above, the impedance parameters (i.e., inertia μ, viscosity η, and stiffness β) of the peripheral arterial wall can be estimated using the two-stage method.

EXPERIMENTS TO VERIFY THE MODEL

To show the validity of the proposed model and proposed impedance estimation algorithm, and to ensure that the index can reduce the effect of factors other than sympathetic nervous activity on changes in arterial wall stiffness, an arm position test[9] was performed under general anaesthesia.

To verify the validity of the proposed method that was described in the previous section, the approximate accuracy of the proposed method could be evaluated using an extracted artery. However, this study adopted a minimally invasive procedure to estimate the arterial characteristics of a living person. To determine the usefulness of the proposed method, the authors evaluated the change in impedance parameters when peripheral blood pressure was forcibly varied during the arm position test, under general anaesthesia.

Figure 2 shows the experimental apparatus of the arm position test. During the experiments, ECG signals, invasive radial arterial blood pressure (IBP), and PPGs of the left thumb were simultaneously measured at 125 Hz, using a bedside monitor (BSS-9800, Nihon Kohden Corp., Tokyo, Japan) with input into a computer via Transmission Control Protocol (TCP).

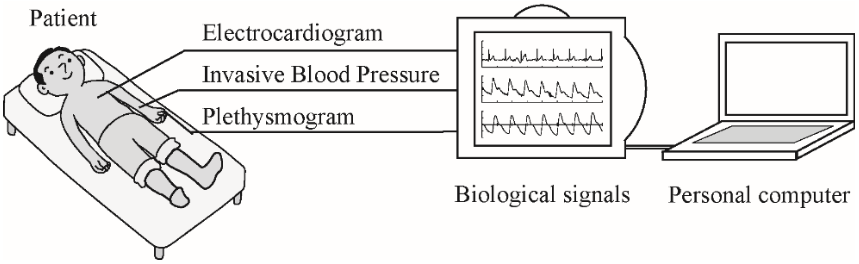

Fig. 2 Experimental instruments.

Figure 3 shows a representative example of ECG, IBP, and PPG. Because the biomedical signals were affected by various artefacts, arterial pressure values and PPGs were pre-processed using digital filters. The ECG signals were filtered out through a second-order infinite impulse response (IIR) band-pass filter (14–28 Hz), and IBP and PPG signals were filtered using a second-order IIR band-pass filter (0.3–10 Hz). Parameters with coefficients of determination of 0.95 or greater between the measured and predicted IBP values were used to assess the patient's condition. Furthermore, outliers caused by disturbances, such as external light and vibration applied to the patient's hands, were excluded before calculation of the proposed index. The threshold level of Equation (13) was defined as the mean IBP for each heartbeat, and was updated in a beat-to-beat fashion.

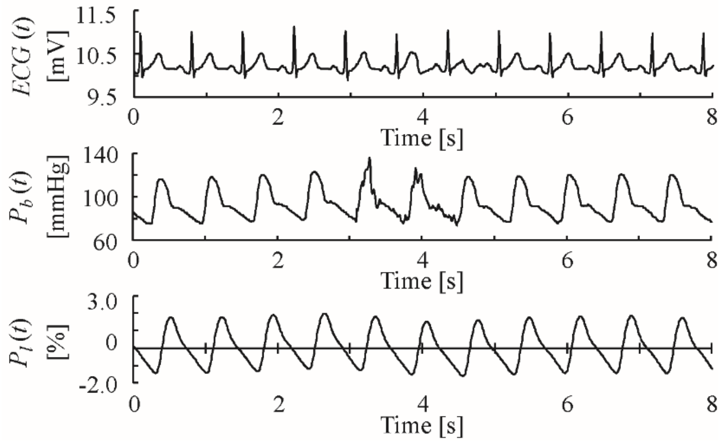

Fig. 3 Example of measured biosignals.

According to the Declaration of Helsinki, informed consent was obtained from all of the patients before the experiments were performed. Approval was obtained from the Hiroshima University Ethics Committee.

In the arm position test, IBP and PPGs, which were associated with intravascular pressure changes in the arm position, were measured, where the fingertip was moved up and down on the basis of the height of the heart. For five patients, simultaneous measurements were performed of invasive radial arterial pressure and PPGs of the thumb on the left side during the arm position test. Patients under general anaesthesia were laid in the supine position on an operating table. The operating table was inclined to the left side by 10–15 degrees, and was tilted two times alternately (Fig. 4). Arm position tests for the patients were performed under general anaesthesia and before operating, as the effect of vasoconstriction by mental tension can have a limited effect.

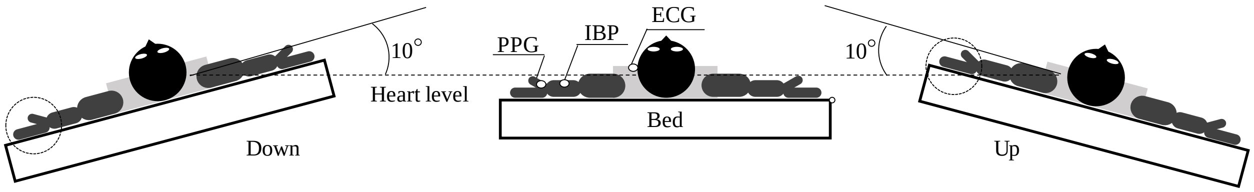

Fig. 4 Arm position test.

We examined the following two possibilities to determine the validity of the proposed method, using biological signal measurements at rest before the arm position test: (i) can the proposed model express the mechanical properties of the peripheral vascular system using stiffness, viscosity, and inertia?; and (ii) can the proposed method estimate the stiffness parameters in the area where the relationship between the log-linearized stiffness arterial pressure component and strain of the arterial diameter is linear[1]?

In (i), three blood pressure components in Equation (6) were determined: (a) measured IBP, (b) residual IBP, following elimination of pressure due to the viscosity component (called viscosity blood pressure, vBP) from the measured IBP, and (c) residual IBP after elimination of vBP and blood pressure due to the inertial component (called inertia blood pressure, iBP) from the measured IBP. The areas of the Lissajous curves that were drawn by the PPG and the IBP components (a), (b), and (c) were calculated; the decreased ratios between (a) and (b), and between (b) and (c), were compared after normalization by the area of the Lissajous curve between the PPG and the measured IBP. Welch's *t*-test was used to determine the significance of differences between (a) and (b), and between (b) and (c), following application of the Bonferroni correction. Differences were considered to be significant for $p < 0.05$. In (ii), the proposed method was verified if indices varied with arm positions. Therefore, the relationship between the log-linearized stiffness IBP component and the PPG is nearly linear in the area containing greater than threshold TH.

VERIFICATION RESULTS

Figure 5 shows an example of the Lissajous curves obtained from Patient A. Figure 6 shows the normalized areas of the Lissajous curves after each patient rested for 20 beats. Figure 6 (a), (b), and (c) correspond to Fig. 5 (a), (b), and (c), where the normalized areas of the Lissajous curves in Fig. 6 (a), (b), and (c) are 1, 0.109 ± 0.044, and 0.029 ± 0.022, respectively. The normalized areas of the Lissajous curves using the residual IBP component after elimination of vBP and iBP were significantly decreased, compared with the normalized areas of the Lissajous curves using IBP components before elimination ([a] vs. [b]: $p = 1.436 \times 10^{-6}$, [b] vs. [c]: $p = 1.152 \times 10^{-2}$).

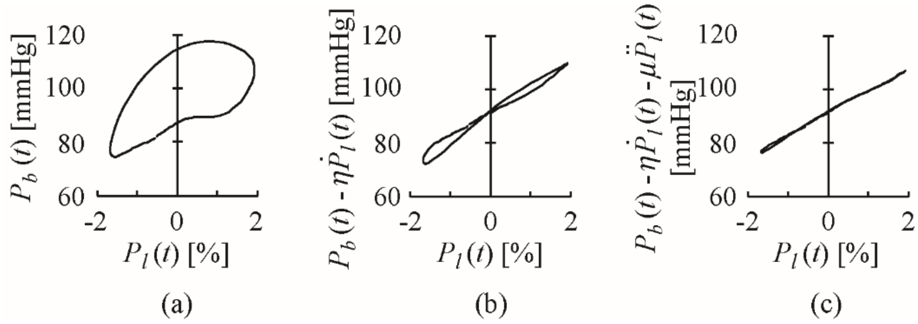

Fig. 5 Representative example of Lissajous curves showing the comparison between IBP and measured $P_l(t)$: (a) $P_l(t)$ vs. $P_b(t)$, (b) $P_l(t)$ vs. $P_b(t) - \eta\dot{P}_l(t)$, (c) $P_l(t)$ vs $P_b(t) - \eta\dot{P}_l(t) - \mu\ddot{P}_l(t)$. The abscissa axis represents the PPG $P_l(t)$ and the vertical axis of (a) represents the measured IBP, that of (b) represents residual IBP after elimination of vBP, and that of (c) represents residual IBP after elimination of viscosity and iBP.

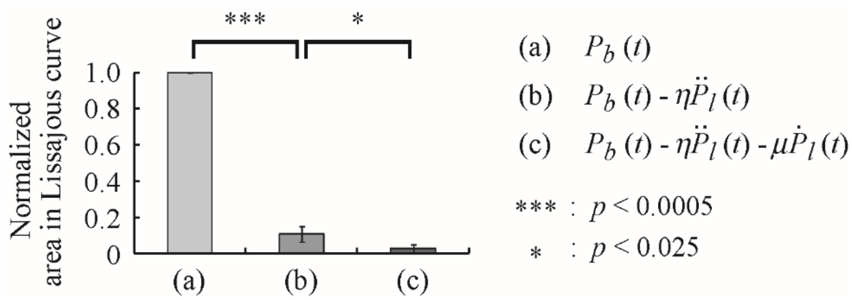

Fig. 6 Comparison of normalized areas in the Lissajous curves.

Figure 7 shows a representative example of the Lissajous curve of the relationship between the PPG $P_l(t)$ and the stiffness-related IBP component, computed from the proposed method. According to Fig. 7, the stiffness-related IBP component computed from the proposed method (broken line) is in good agreement with the log-linearized stiffness IBP component of the proposed model (solid line) under the area where IBP was higher than or equal to the threshold TH (i.e., $P_l(t) \geq 0$). However, the broken line was curved and greatly differed from the solid line at the area where the measured IBP was lower than the threshold TH (i.e., $P_l(t) < 0$). The difference between the broken and solid lines corresponds to the nonlinear characteristic $P_{b_{\beta_{nl}}}(P_l(t))$ of Equation (6). Mean square errors were calculated between the broken and solid lines for the same beats shown in Fig. 7. Figure 8 shows the results for the average values of all patients, in the areas where IBP was higher than or equal to and lower than the threshold TH. The mean square error was $5.37 \times 10^{-2} \pm 2.62 \times 10^{-2}$ and $1.13 \times 10^{-2} \pm 0.77 \times 10^{-2}$ at the area where the blood pressure was higher than or equal to the threshold TH, and was less than the threshold TH, respectively; a significant difference was present between the two areas ($p = 1.99 \times 10^{-2}$).

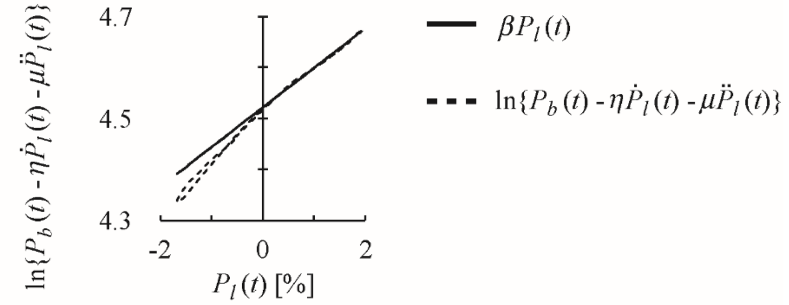

Fig. 7 Relationships between stiffness-related IBP and measured $P_l(t)$. The solid line represents the log-linearized stiffness IBP component of the proposed model. The broken line represents the stiffness-related IBP component computed from the proposed method.

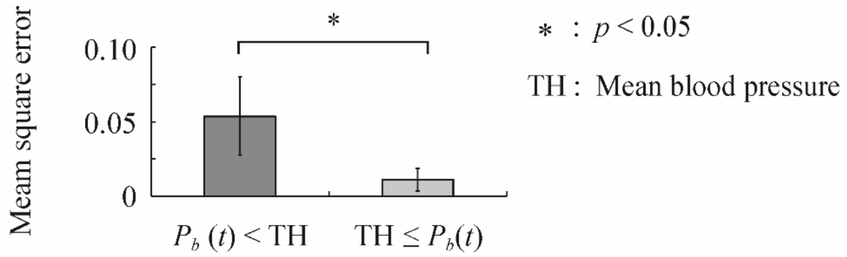

Fig. 8 Mean square error between predicted IBP by the stiffness $\beta P_l(t)$ and the logarithm of residual IBP in $P_b(t) - \eta P_b(t) - \ddot{P}_l(t)$.

REFERENCES

1. Hirano, H., *et al*. A Log-linearized peripheral arterial viscoelastic index and its application to endoscopic thoracic sympathectomy. *Trans. Soc. Instr. Control Engineers* **48**, 731–739 (2012).

2. Hirano, H., *et al*. Monitoring of peripheral vascular condition using a log-linearized arterial viscoelastic index during endoscopic thoracic sympathectomy. *Conf. Proc. IEEE. Eng. Med. Biol. Soc.* **2013**, 2587–2590 (2013).

3. Armentano, R.L., Barra, J.G., Levenson, J., Simon, A. & Pichel, R.H. Arterial wall mechanics in conscious dogs: assessment of viscous, inertial, and elastic moduli to characterize aortic wall behavior. *Circ. Res.* **76**, 468–478 (1995).

4. Barra, J.G., *et al*. In vivo angiotensin angiotensin II receptor blockade and converting enzyme inhibition on canine aortic viscoelasticity. *Am. J. Physiol.* **272**, H859–H868 (1997).

5. Nagasawa, S., *et al*. Mechanical properties of canine femoral artery smooth muscle. *J. Jpn. Coll. Angiology*. **20**, 221–226 (1980).

6. Hayashi, K., Handa, H., Nagasawa, S., Okumura, A. & Moritake, K. Stiffness and elastic behavior of human intracranial and extracranial arteries. *J. Biomechanics* **13**, 175–184 (1980).

7. Aoyagi, T. & Ukawa, T. Pulse oximeter which the optical measurement produced. Structure and principle of a pulse oximeter. *Clin. Eng*. **7**, 102–110 (1996).

8. Ukawa, T. Pulse oximeter. *J. Med. Lab*. **22**, 11–16 (1994).

9. Takemiya, T., Maeda, J., Suzuki, J., Nishihira, Y. & Shimoda, M. Differential digital photoplethysmographic observations of finger vascular exponential response to the arm position changes in humans. *Adv. Exercise Sports Physiol*. **2**, 83–90 (1996).
